# Supplementary material for: Genome-Wide Identification, Characterization, and Expression Analysis of the BES1 Family Genes under Abiotic Stresses in Phoebe bournei
Source: Int J Mol Sci. 2024 Mar 6;25(5):3072. doi: 10.3390/ijms25053072 (PMC10932303; doi:10.3390/ijms25053072)
Supplement: Supplementary file 1 [file ijms-25-03072-s001.zip › Table captions.pdf]

Table S1. The expression of the 9 *PbBES1* genes in various tissues, including root bark, root xylem, stem bark, stem xylem, and leaf.
Table S2. Information of primers.
